# Transduction of Craniofacial Motoneurons Following Intramuscular Injections of Canine Adenovirus Type-2 (CAV-2) in Rhesus Macaques

**DOI:** 10.3389/fnana.2019.00084

**Published:** 2019-09-18

**Authors:** Martin O. Bohlen, Hala G. El-Nahal, Marc A. Sommer

**Affiliations:** ^1^Department of Biomedical Engineering, Duke University, Durham, NC, United States; ^2^Department of Neurobiology, Duke University School of Medicine, Durham, NC, United States

**Keywords:** canine adenovirus type-2 (CAV-2), adenovirus, motoneurons, oculomotor, monkey, primate, gene therapy

## Abstract

Reliable viral vector-mediated transgene expression in primate motoneurons would improve our ability to anatomically and physiologically interrogate motor systems. We therefore investigated the efficacy of replication defective, early region 1-deleted canine adenovirus type-2 (CAV-2) vectors for mediating transgene expression of fluorescent proteins into brainstem motoneurons following craniofacial intramuscular injections in four rhesus monkeys (*Macaca mulatta*). Vector injections were placed into surgically identified and isolated craniofacial muscles. After a 1- to 2-month survival time, animals were sacrificed and transgene expression was assessed with immunohistochemistry in the corresponding motoneuronal populations. We found that injections of CAV-2 into individual craniofacial muscles at doses in the range of ∼10^10^ to 10^11^ physical particles/muscle resulted in robust motoneuronal transduction and expression of immunohistochemically identified fluorescent proteins across multiple animals. By using different titers in separate muscles, with the resulting transduction patterns tracked via fluorophore expression and labeled motoneuron location, we established qualitative dose-response relationships in two animals. In one animal that received an atypically high titer (5.7 × 10^11^ total CAV-2 physical particles) distributed across numerous injection sites, no transduction was detected, likely due to a retaliatory immune response. We conclude that CAV-2 vectors show promise for genetic modification of primate motoneurons following craniofacial intramuscular injections. Our findings warrant focused attention toward the use of CAV-2 vectors to deliver opsins, DREADDs, and other molecular probes to improve genetics-based methods for primate research. Further work is required to optimize CAV-2 transduction parameters. CAV-2 vectors encoding proteins could provide a new, reliable route for modifying activity in targeted neuronal populations of the primate central nervous system.

## Introduction

Replication defective, early region 1-deleted canine adenovirus type 2 (CAV-2) vectors are an increasingly important tool for neurological research due to CAV-2’s neuronal tropism and capacity to retrogradely label neurons projecting to the injection sites. This is particularly true for non-human primate research, where CAV-2 has increasingly been used for anatomical investigations and perturbation of neuronal circuits.

Early work found that CAV-2 efficiently and preferentially transduces to cultured motoneurons. CAV-2-GFP injected into the gastrocnemius of newborn mice produced motoneuronal labeling within the ventral horn of the lumbosacral spinal cord 24-days post-injection (Soudais et al., 2001). This and other work suggested that CAV-2 binds to the coxsackie and adenovirus receptor (CAR) (Soudais et al., 2000, 2001; Loustalot et al., 2016). Injections into the tibialis anterior, gastrocnemius, and diaphragm muscles of mice resulted in no transduction to the muscle fibers but did yield transduction in the motoneurons (Soudais et al., 2001; Salinas et al., 2009). This is likely because in mature, non-pathological (i.e., normal) muscles, CAR expression is confined to neuromuscular junctions (Shaw et al., 2004; Sinnreich et al., 2005).

In an elegant series of experiments, Salinas et al. (2009) provided compelling evidence that, in cell culture, once bound to CAR, CAV-2 undergoes rapid clathrin-mediated formation endosomes in the first 5 min. At the motoneuronal terminals, once internalized, Salinas and colleagues observed a ∼30-min period where the intracellular CAV-2 containing endosomes oscillated locally at the region of internalization, before undergoing long-range retrograde axonal transportation. In the same series of experiments, injections of CAV-2 into the tibialis anterior and gastrocnemius muscles of mice revealed the presence of internalized CAV-2 positive endosomes in axons of the sciatic nerve following an 8-h survival period (Salinas et al., 2009).

In addition to CAV-2’s efficacy at transducing motoneurons via injections into muscles, there is a growing body of evidence demonstrating its utility when injected into brain areas. Intracerebral injection of CAV-2 labels neurons both locally at the injection site and retrogradely in areas that project to the injection site (reviewed in Box 1 of Del Rio et al., 2019). In primates, CAV-2 has proven effective for transducing genes into multiple regions of the central nervous system, for example in the Cd of *Microcebus murinus* using helper-dependent CAV-GFP (Mestre-Frances et al., 2018).

Given the accruing evidence for CAV-2’s efficacy as a vector, our goal was to test the ability of CAV-2 to retrogradely label primate motoneurons following intramuscular craniofacial injections, with a focus on the extraocular musculature. The extraocular muscles, in particular, provide a unique and sensitive testbed for characterizing viral vectors for their capacity to deliver their genomic payload to targeted cellular populations, for two reasons. First is their unique pattern of innervation. The extraocular muscles receive two patterns of innervation: 80–90% of the muscle fibers receive nervous input at a single point in the middle third (or belly) of the muscle, while the remaining muscle fibers receive multiple neuromuscular junctions from the origin of the muscle fiber at the back of the orbit through to the muscle’s insertion into the globe (Spencer and Porter, 1988, 2006). This unique innervation pattern provides the opportunity to target injections to separate classes of fibers within the same muscle. Second, in primates, the neurons forming the single neuromuscular junctions, called singly innervating fiber or “SIF” motoneurons, are found within the cytoarchitectonic boundaries of the three extraocular motor nuclei. In contrast, the neurons forming the multiple neuromuscular junctions, called multiply innervating fiber or “MIF” motoneurons, are found in the periphery of the respective three extraocular motor nuclei. *Oculomotor nucleus*: The medial and inferior rectus motoneurons cap the oculomotor nucleus by sitting on its dorsomedial periphery; this division has been termed the C-group (Büttner-Ennever et al., 2001; Tang et al., 2015). Also, the MIF motoneurons of the inferior oblique and superior rectus sit sandwiched between the two oculomotor nuclei, on the midline; this division of these motoneuronal pools has been termed the S-group (Büttner-Ennever et al., 2001). *Trochlear nucleus*: Superior oblique MIF motoneurons sit on the dorsal cap of the trochlear nucleus. *Abducens nucleus*: MIF motoneurons of the lateral rectus sit in the ventral, medial and dorsal peripheries of the abducens nucleus (Büttner-Ennever et al., 2001). Together, these two features of extraocular motor units – unique patterns of muscle innervation and distinct motoneuronal pools – make the oculomotor system favorable for characterizing viral vectors and optimizing techniques of intramuscular viral injections.

The parameters of most interest to us were injection location and the propensity for a vector to diffuse from the injection site. Both are critical parameters to consider, as the proximity of the vector to the neuromuscular junction may play a critical role in determining whether an injected vector is able to successfully transduce motoneuronal terminals. In the case of *in vivo* muscle injections, the ∼25 nm AAV (Colella et al., 2018) or ∼100 nm CAV-2 capsids (Schoehn et al., 2008) must diffuse from the injection site through the tightly organized intramuscular and extracellular spaces to reach their receptors on the motoneuronal terminals. During the interval between depositing the vectors into the muscle and the vector binding to its receptor, the vector must survive an immunological gauntlet that is initiated as soon as the needle is inserted. The act of injecting the muscle causes trauma and extravasation of several immune system agents (Tidball, 2017; Sass et al., 2018). To improve their odds of motoneuronal transduction, Williams et al. (2018) performed low-threshold electrical stimulation of skeletal muscles in three macaques. They assumed that areas in which the largest contractions were elicited following the smallest stimulation parameters were the sites of neuromuscular junctions, and injected adeno-associated virus, serotype 6 (AAV6) at these locations. This resulted in muscle fasciculations when the peripheral nerves were optically illuminated. This technique has yet to be tested with CAV-2.

Here we analyzed the capacity of CAV-2 to transduce fluorescent proteins into motoneurons following craniofacial intramuscular injections in rhesus macaques. In three of four animals tested, CAV-2 transduced its genes into motoneurons reliably. In general, efficacy was a function of dose (number of particles). The results confirm the efficacy of CAV-2 for transduction of cranial motoneurons in primates and illustrate the usefulness of the uniquely innervated extraocular muscles as a testbed for viral transduction.

## Materials and Methods

### Animals

Four rhesus macaques (*Macaca mulatta*) were included in this investigation (Table 1). All procedures were in accordance with the NIH Guide for the Care and Use of Laboratory Animals and approved by the Duke University IACUC. Some animals received additional intraparenchymal injections that are not reported here. All animals included in the current report received additional viral injections placed in the other extraocular muscles. These injections included different serotypes of adeno-associated virus (AAV), herpes simplex virus (HSV) and lentivirus. No other adenovirus was tested besides CAV-2.

**TABLE 1:** Case information for the animals.

**Animal**	**Age**	**Sex**	**Survival Duration**	**Weight**	**Total CAV-2**
	**(Years)**		**(Days)**	**(kgs)**	**Particles Injected**
M18-01	19	M	23	10.2	5.2 × 10^10^
M18-02	18	M	63	8.6	5.7 × 10^11^
M18-03	10	F	60	5.6	4.2 × 10^11^
M19-01	11	F	50	8.9	1.6 × 10^11^

### Viral Vectors

All CAV-2 vectors came from the Platform de Vectorology de Montpellier (PVM). Viruses were shipped in dry ice and stored at −80°C until they were used. To minimize the number of freeze-thaws, aliquots were made following the first thaw for an injection, rather than upon receipt of the stock virus. Table 2 lists the vectors used and relevant parameters of the injection procedures. To test dose responses, custom titers of CAV-2 were created by diluting stock titers with Dulbecco’s phosphate buffered saline (MilliporeSigma, St. Louis, MO, United States; D8537/MDL number: MFCD00131855).

**TABLE 2:** Injection information.

**Animal**	**Vector**	**Stock Titer**	**No. CAV-2 Particles**	**Injection Volume**	**Injection**	**Degree of Motoneuron**
		**(pp/μl)**	**Injected**	**(μl)**	**Location**	**Labeling**
M18-01	CAV-2-CMV-mCitrine	2.9 × 10^9^	5.2 × 10^10^	18	ObOc	++++
M18-02	CAV-2-CMV-mCitrine	2.9 × 10^9^	7.2 × 10^10^	25	SR	0
	CAV-2-CMV-DsRedII	5.4 × 10^9^	2.7 × 10^10^	25	MR	0
	CAV-2-CMV-DsRedII	5.4 × 10^9^	7.0 × 10^10^	25	LR	0
	CAV-2-CMV-mCitrine	2.9 × 10^9^	3.0 × 10^10^	15	ObOc	0
	CAV-2-hChAT-GFP	1.0 × 10^10^	4 × 10^11^	40	M	0
M18-03	CAV-2-CMV-mCitrine	2.9 × 10^9^	1.4 × 10^10^	15	ObOc	+
	CAV-2-CMV-mCitrine	6.5 × 10^9^	7.8 × 10^10^	12	SR	++
	CAV-2-hChAT-GFP	1.0 × 10^10^	2.4 × 10^11^	24	IR	Inconclusive
	CAV-2-CMV-DsRedII	5.4 × 10^9^	6.2 × 10^10^	22	LR	+
	CAV-2-CMV-DsRedII	5.4 × 10^9^	2.4 × 10^10^	22	MR	+
M19-01	CAV-2-CMV-DsRedII	5.4 × 10^9^	1.1 × 10^11^	20	MR	+++
	CAV-2-CMV-DsRedII	5.4 × 10^9^	5.6 × 10^10^	20	LR	++

### Surgical Procedures

Dexamethasone (2.0 mg/kg, IM) or Solu-Medrol (15.0 mg/kg, IM) was administered 24 h prior to surgery and immediately before surgery for mild immunosuppression. Animals were sedated with ketamine hydrochloride (3.0 mg/kg, IM) and dexdomitor (0.075 mg/kg, IM), then a surgical plane of anesthesia was maintained using 1–3% isoflurane and oxygen. All surgical procedures were carried out under aseptic conditions using sterile techniques.

#### Supraorbital Approach

Orbicularis oculi and superior rectus muscle injections required a surgical approach from the superior orbit. Animals were first placed into a stereotaxic frame (Kopf Instruments, Tujunga, CA, United States) and 0.5–1.0 ml of 0.25% bupivacaine was administered cutaneously along a planned incision line on the supraorbital ridge. Next, the incision was made, forming a small tissue flap that revealed the supraorbital insertion point of the annular orbicularis oculi. A second incision was made just inferior to the insertion point to reveal the superior retroorbital space and provide access to the levator palpebrae superioris, superior rectus, and superior oblique muscles. Blunt dissection was used to identify and isolate each muscle. Once isolated, muscles were looped with suture to allow for clean, precise injections. Injections were made by pushing a sharp needle attached to an appropriately sized Hamilton syringe (10–100 μl) through the insertion, toward the belly of each muscle. As the needle traversed the muscle, care was taken to avoid exiting the epimysium with the needle point. Once the needle point was in position, small bolus injections were made along the length of the needle track as it was slowly retracted from the muscle. In larger volume injections, the muscle expanded noticeably as the viral vector solution was dispensed along the injection track. To inject the orbicularis oculi, a very thin muscle that sits inside the eyelid, a sharp Hamilton syringe was inserted into the lateral aspect of the tissue flap and pushed medially through the lid. Once again, small bolus injections were placed along the length of the needle track as it was slowly withdrawn. Following injections, the retroorbital space was thoroughly washed with sterile saline. The orbicularis oculi was sutured back to its supraorbital insertion and the dermal incision was reapproximated and sutured along the supraorbital ridge.

#### Conjunctival Approach

Muscles in the inferior half of the orbit were approached via the conjunctiva. Animals were placed on their back and a small incision was made through the conjunctiva near the corneal junction around the bottom two thirds of the eye. Tissues were blunt dissected to reveal the insertion points of the three targeted muscles: the medial, lateral and inferior recti. These muscles were injected using the same procedure described above. Once injections were made, the site was flushed with sterile saline, the conjunctiva was reapproximated, and ophthalmic ointment was applied to the eye. No sutures were necessary as the conjunctiva heals without them.

### Masseter

For masseter muscle injections, the surgeon digitally located the muscle belly. The overlying skin was then injected with 0.5 ml of 0.25% bupivacaine and then a small incision was made to reveal the muscle. Viral injections were made into the masseter as described above, then dermal layers were reapproximated and sutured.

At the end of surgery, a second cutaneous injection (0.5–1.0 ml) of 0.25% bupivacaine was administered along incision lines. Before exiting the surgical suit, all personnel removed their personal protective equipment (PPE) and sprayed their shoe-bottoms with RescueTM (Virox Technologies Inc., Oakville, ON, Canada). Postoperatively, animals received buprenorphine SR (0.2 mg/kg, IM) for analgesia. Additionally, either dexamethasone (1.0 mg/kg, IM for 3 days, then 0.5 mg/kg, IM every other day for 7 days) or Solu-Medrol (15.0 mg/kg, IM for 2 days, then 10.0 mg/kg, IM for 3 days, then 5.0 mg/kg, IM for 2 days, then 1.0 mg/kg, IM for 3 days) was administered. Post-operatively, animals were housed in an isolated room for a minimum of 48 h to prevent any potential shedding of vectors that could sensitize naïve animals to the viruses being used. Experimenters, staff, and veterinarians that entered the colony room housing the injected animals were not allowed to enter any other colony rooms for 12–48 h. Personnel removed all PPE before leaving the colony room, immediately sprayed down their shoes with RescueTM outside of the room, and thoroughly washed their hands with soap and water. Following isolation, animals were returned to their home colony room but were not allowed access to any other animals in the room unless the cage-mate had already received viral injections. In general, the injected animals were always the last to be worked with during the day, and if there was a need to go back to work with naïve animals, PPE was changed. Post-operative recovery was uneventful and, during the survival periods, no symptoms of neural deficits were observed.

### Histology

After viral injections, animals survived long enough to allow for transduction and subsequent expression of fluorescent proteins (23–63 days, Table 1). Then we performed euthanasia and perfusion, tissue preparation, and immunohistochemistry.

#### Euthanasia and Perfusion

At the end of the survival period, animals were sedated with ketamine hydrochloride (3.0 mg/kg, IM) and then administered a lethal dose of pentobarbital (50.0 mg/kg, IP). Animals were then transcardially perfused with 4 L of chilled 0.1 M, pH 7.4 phosphate buffered saline (PBS), followed by 4 L of 4% paraformaldehyde in 0.1 M, pH 7.4 PBS.

#### Tissue Preparation

After perfusion, the brain was extracted, stereotaxically cut into blocks, and post-fixed in 4% paraformaldehyde in 0.1 M, pH 7.4 PBS at 4°C overnight. Blocks were then cryoprotected in PBS containing 30% sucrose at 4°C with agitation. Once sunk, blocks were cut into 75-μm-thick coronal sections using a freezing stage sliding microtome (American Optical Company, Buffalo, NY, United States) and stored in PBS at 4°C. For processing, sections were divided into 6 rostral to caudal series, with ∼450 μm between adjacent sections in the series.

#### Immunohistochemistry

Due to variability in the intensity and quality of signal that was provided through unaided epifluorescence across cases, and to avoid fading of the fluorescent signal over time, we opted to perform immunohistochemical amplification. This provided a standardized method for visualizing the degree of intracellular labeling, while also establishing a fade-resistant anatomical signal. Serial, free-floating sections were rinsed in 0.1 M, pH 7.4 PBS then, to block endogenous peroxidase activity, they were placed in 0.3% H_2_O_2_ in PBS solution. Following a second PBS wash, sections were placed in a solution of 0.25% Triton X-100 in PBS. Afterward, sections were moved to a 1% BSA/0.25% Triton X-100 in PBS solution to prevent non-specific antibody binding. Sections were then ready for incubation with primary antibody.

##### Anti-green fluorescent protein (GFP)/mCitrine

Despite differences in spectral characteristics, mCitrine and GFP have very high amino acid sequence homology which causes primary antibodies against GFP to also bind to YFP family proteins (including mCitrine). Therefore, to detect CAV-2 mediated expression of mCitrine, sections were incubated in a solution of biotinylated goat anti-GFP (1:1000, Rockland Immunochemicals Inc., Limerick, PA, United States; 600-106-215) in 1% BSA/0.25% Triton X-100 in PBS for 1–3 h at room temperature, then ∼48 h at 4°C with agitation. Sections were then rinsed with PBS and incubated with biotinylated rabbit anti-goat IgG secondary antibody (Vector Laboratories, INC., Burlingame, CA, United States; PK6105) for 1.5 h at room temperature.

##### Anti-DsRedII

For visualization of the RFP family of fluorophores, sections were incubated in rabbit anti-RFP conjugated to horseradish peroxidase (1:300, Rockland Immunochemicals Inc., Limerick, PA, United States; 600-403-379) or rabbit anti-RFP (1:300, Rockland Immunochemicals Inc., Limerick, PA, United States; 600-401-379) in a 1% BSA/0.25% Triton X-100 in PBS solution for 1–3 h at room temperature, then ∼48 h at 4°C. Next, sections were washed with PBS and incubated in biotinylated goat anti-rabbit IgG secondary antibody (Vector Laboratories, Inc., Burlingame, CA, United States; PK-6101) for 1.5 h at room temperature.

After antibody treatment, sections were transferred to an avidin-biotin-horseradish peroxidase complex (ABC) solution (Vector Laboratories, Inc., Burlingame, CA, United States; PK6105) for 1 h at room temperature. Sections were then rinsed with PBS and placed in 0.5% DAB/0.01% cobalt chloride/0.01% nickel ammonium sulfate in PBS solution for 20 min. Finally, to catalyze the reaction, 0.3% H_2_O_2_ was added to the solution. This produced a brown-black product in structures expressing the transgene. After a final PBS wash, sections were mounted to glass slides and allowed to dry overnight. Sections were then counterstained with thionin and dehydrated with graded ethanols then xylene. Sections were coverslipped using Cytoseal 60 (Thermo Fisher Scientific, Waltham, MA, United States).

### Analyses

Low magnification drawings were made using a Bausch & Lomb projection microscope (Rochester, NY, United States). 10x medium and 100x high magnification drawings were made using drawing tubes mounted to a Zeiss Axioskop. Drawings were vectorized using CorelDRAW 2018. Digital photomicrographs were taken on a Keyence BZ-X800 (Itasca, IL, United States) affixed with a digital color camera, operated using Keyence BZ-H4XD software. When necessary, images were adjusted for brightness, contrast and color using Adobe Photoshop CS8 to provide the most accurate representation of how samples appeared under the microscope.

## Results

**FIGURE 1:**
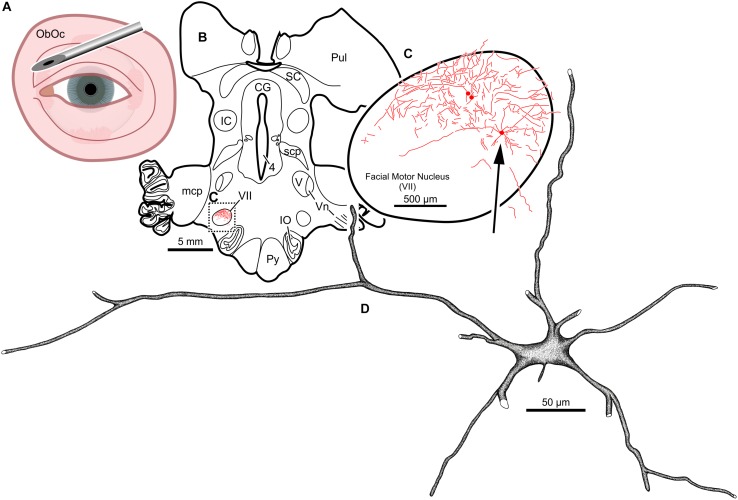
Facial motoneuronal labeling following injection of CAV-2-CMV-mCitrine into the left orbicularis oculi. **(A)** Approximate angle and location of the orbicularis oculi injection **(B)** Low magnification view of the coronal section that contained the left facial motor nucleus, box in **(A)** indicates location of drawing shown in **(B)**. **(C)** A medium magnification view of the facial motor nucleus. CAV-2 mediated motoneuronal labeling was observed throughout the dorsomedial division of the facial motor nucleus. **(D)** A high magnification, camera lucida reconstruction of a single neuron from this case demonstrating the intracellular distribution of mCitrine expression. CAV-2 transgene expression resulted in the ability to visualize the somata, primary and secondary dendrites in addition to axonal labeling (*not illustrated*).

**FIGURE 2:**
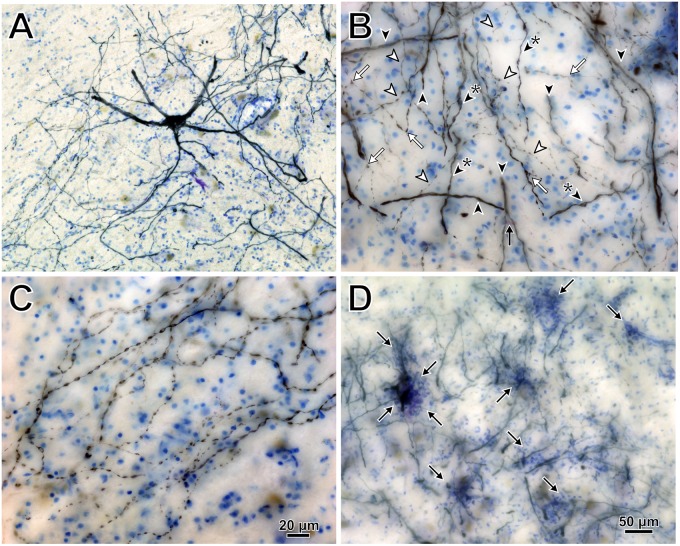
Photomicrographs of CAV-2 mediated retrogradely labeled orbicularis oculi motoneuronal somata, dendrites and axons from the same case presented in Figure 1. **(A)** View of CAV-2 mediated motoneuronal labeling within the facial motor nucleus. **(B)** Labeling ventral to the dorsomedial division of the facial motor nucleus. **(C)** A large number of orbicularis oculi motoneuronal projections with varicosities were observed coalescing in the ventrolateral portion of the facial motor nucleus. **(D)** Low magnification view showing the distribution of glia (light blue) within the dorsomedial division of the facial motor nucleus; areas enriched with glia are highlighted with arrows. Photomicrographs **(A,D)** were taken at 20x, while **(B,C)** were taken at 40x. A = 12z, B = 9z, and D = 7z.

Intramuscular injections of CAV-2 were made into different craniofacial muscles of four rhesus macaques with the intent of assessing the vector’s capacity for reliable uptake at the terminals of cranial nucleus motoneurons. In the first case, an 18 μl injection of CAV-2-CMV-mCitrine (5.2 × 10^10^ pp) was made into the left orbicularis oculi (Figure 1A). This was the only CAV-2 injection made in this animal, “M18-01” (Table 2). The injection resulted in fairly robust labeling of motoneurons in the dorsomedial division of the left facial motor nucleus (VII; Figures 1B,C). In addition to dense dendritic labeling, several labeled somata were observed (Figure 1C; example motoneuron in Figure 1D). CAV-2 retrogradely labeled soma and primary and secondary dendrites with some regularity. Figure 2 shows photomicrographs from a neighboring section from the same case. A medium magnification photomicrograph (Figure 2A) shows an example labeled neuron and illustrates the density of dendritic labeling within the dorsomedial division of the facial motor nucleus. A higher magnification photomicrograph of the ventral labeling from the same section as in Figure 2A revealed labeled neuronal structures with numerous varicosities that were partitioned by fine filaments (Figure 2B, white arrowheads). In some regions, for example directly ventral toward the central core of the facial motor nucleus, a number of 90° bifurcations were observed (Figure 2B, white arrows). In this same region, there were a number of labeled neuronal structures presumed to be dendrites (Figure 2B, black arrow heads). In some instances, there were labeled neuronal structures that presented with morphology reflecting mildly beaded dendrites (Figure 2B, black arrowheads with a “^∗^”). Finally, a presumed acutely angled dendritic branch is highlighted (Figure 2B, black arrow). In other regions, for example the ventrolateral portions of the facial motor nucleus, there were similar labeled morphological features with minimal branching (Figure 2C). Finally, we noted some gliosis in this case (Figure 2D). The dorsomedial division of the facial motor nucleus exhibited a marked increase in the presence of glia (Figure 2D, black arrows), suggesting a degree of neurotoxicity.

In the next animal, we attempted to study the dose-response relationship by injecting four different doses of CAV-2-CMV and one dose of CAV-2-hChAT into the left orbital musculature (“M18-02”; Tables 1, 2). This animal received a total of 5.7 × 10^11^ pp of CAV-2, in addition to other injections placed intramuscularly and intraparenchymally (data not shown). The animal showed no transduction across all injections, which we suspect was due to an exaggerated immune response to the high viral load (see Discussion). The animal nevertheless remained asymptomatic through the survival period, exhibiting normal behavior and no overt signs of an immune reaction.

**FIGURE 3:**
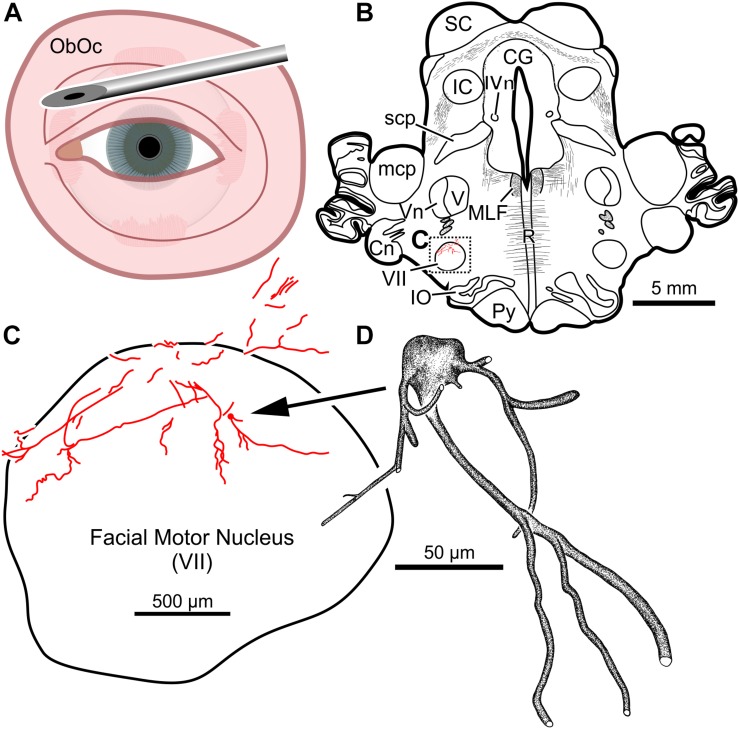
Facial motoneuronal labeling following an injection of CAV-2-CMV-mCitrine into the left orbicularis oculi. **(A)** Approximate angle and location of orbicularis oculi injections. **(B)** Low magnification view of a representative section containing the left facial motor nucleus, box in **(B)** indicates location of drawing shown in **(C)**. **(C)** A medium magnification view of the facial motor nucleus. In this case, CAV-2 injected into the orbicularis oculi provided retrograde labeling, albeit to a lesser extent when compared to the first case. **(D)** A high magnification, camera lucida reconstruction of a single neuron from this case demonstrating the intracellular distribution of mCitrine expression of a single cell.

In the third animal, five injections into the orbital musculature were made again, but to decrease the presumed immune response, the total number of viral particles was reduced to 4.2 × 10^11^ (“M18-03”; Tables 1, 2). First, 15 μl of CAV-2-CMV-mCitrine (1.4 × 10^10^ pp) injected into the left orbicularis oculi (Figure 3A) produced retrograde labeling of facial motor nucleus motoneurons (Figures 3B,C). There was a clear reduction both in the density of dendritic labeling and number of labeled soma compared to the first case (cf. Figure 1C), but the labeling was robust enough to allow visualization of somata, primary, secondary, and tertiary dendrites, while axonal labeling was sparse (Figure 3D).

**FIGURE 4:**
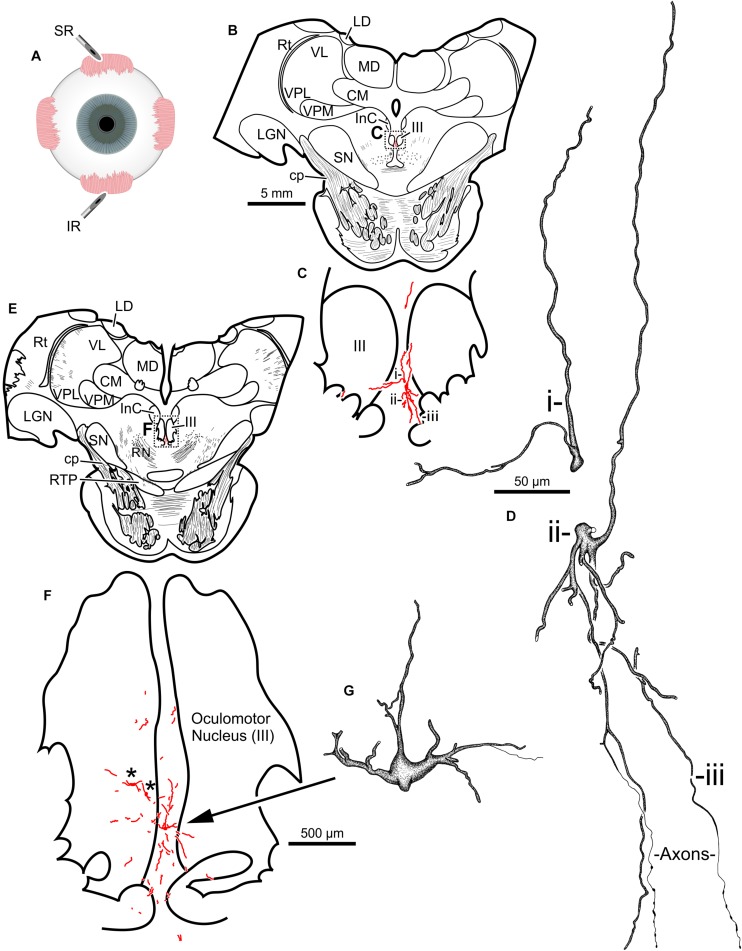
An injection of CAV-2-CMV-mCitrine into the left superior rectus **(A)** resulted in motoneuronal labeling along midline, in the region of the S-group, between the left and right oculomotor nucleus. Low magnification views from representative sections containing the rostral **(B)** and more caudal **(E)** oculomotor nucleus, box in **(B)** indicates location of drawing shown in **(C)**, box in **(E)** indicates the location of the drawing shown in **(F)**. **(C,F)** Medium magnification views of the oculomotor nucleus and the distribution of superior rectus motoneuronal labeling within. ^∗^ in **(F)** indicates potential inferior rectus motoneurons labeled in the left oculomotor nucleus. **(D,G)** High magnification, camera lucida reconstructions of single neurons from this case, demonstrating the intracellular distribution of mCitrine expression within single cells. Labeled neuronal structures (i, ii, and iii) in **(C)** are presented as high magnification in **(D)**. Magnifications are the same for **(B,E)**, **(C,F)** and **(D,G)**.

The second injection in this animal (M18-03) consisted of 12 μl CAV-2-CMV-mCitrine (7.8 × 10^10^ pp) placed into the left superior rectus muscle (Figure 4A). This produced retrograde labeling of superior rectus motoneurons, primarily on the midline between the two oculomotor nuclei (Figures 4B,C,E,F) which is known to be the territory of S-group MIF motoneurons of the superior rectus muscle. This injection resulted in the largest number of retrogradely labeled motoneurons in this animal. In addition to labeled somata, there were many labeled, short dendritic shoots running through the sections. Careful inspection of midline motoneuronal morphology, in known loci for superior rectus but not inferior rectus MIF motoneurons, revealed frequent labeling of the soma as well as primary, secondary and tertiary dendrites. Labeled axons were often observed exiting the oculomotor complex ventrally and could be observed within the fascicles of the oculomotor nerve (Figures 4D,G). This series also showed motoneuronal labeling within the oculomotor nucleus ipsilateral to the injected orbit (Figure 4F, indicated by ^∗^).

Third, 24 μl of CAV-2-hChAT-GFP (2.4 × 10^11^ pp) was injected into the left inferior rectus (Figure 4A) to test the hChAT promotor for its ability to provide transgene expression in motoneurons. The primary antibody, goat anti-GFP, is unable to discriminate between GFP and mCitrine labeling. The motoneuronal labeling in the left oculomotor nucleus, however, suggested that the hChAT promoter may have been effective.

**FIGURE 5:**
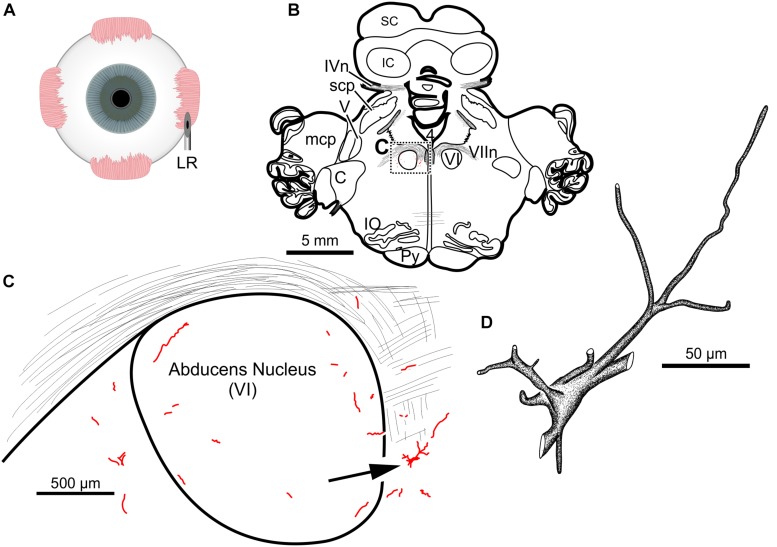
An injection of CAV-2-CMV-DsRedII into the left lateral rectus **(A)** resulted in motoneuronal labeling predominantly in the periphery of the left abducens nucleus. **(B)** Low magnification view from a representative section containing the abducens nucleus, box in **(B)** indicates location of drawing shown in **(C)**. **(C)** A medium magnification view of the abducens nucleus and the distribution of lateral rectus motoneuronal labeling predominantly in the surround of the left abducens nucleus. **(D)** High magnification, camera lucida reconstruction of a single neuron from this case demonstrating the extent of intracellular DsRedII labeling expression within a single cell.

The fourth injection in the same animal consisted of 22 μl of CAV-2-CMV-DsRedII (6.2 × 10^10^ pp) into the left lateral rectus muscle (Figure 5A). This resulted in retrograde labeling of lateral rectus motoneurons in the periphery of the left abducens nucleus (Figures 5B,C), a location attributed to MIF motoneurons of the lateral rectus in primates (Büttner-Ennever et al., 2001). While this injection produced more neuronal labeling compared to the lowest titer injection into the medial rectus from the same case, there was less viral transgene expressed from this injection compared to the higher titer injections in this animal (M18-03; Figure 5C compared to Figures 4C,F) and the animal that received a single CAV-2 injection (M18-01; Figure 1C). This injection labeled soma and primary, secondary and tertiary dendrites (Figure 5D).

**FIGURE 6:**
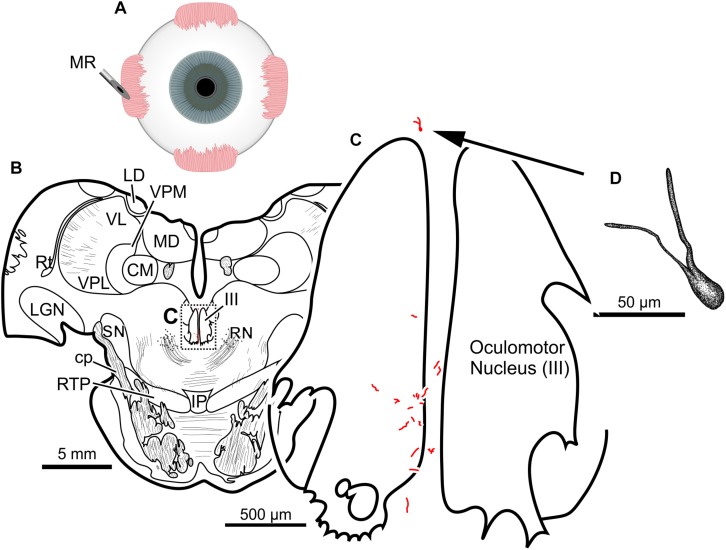
An injection of CAV-2-CMV-DsRedII into the left medial rectus **(A)** resulted in motoneuronal labeling both within and outside the left oculomotor nucleus. **(B)** Low magnification view from a representative section containing the oculomotor nucleus, box in **(B)** indicates location of drawing shown in **(C)**. **(C)** A medium magnification view of the oculomotor nucleus and the distribution of medial rectus motoneuronal labeling within and to a lesser extent, outside the cytoarchitectonic boundaries of the left oculomotor nucleus. **(D)** High magnification, camera lucida reconstruction of a single neuron from this case demonstrating the intracellular distribution of DsRedII expression within a single cell. Based on its location, this neuron is a presumed medial rectus multiply innervating fiber motoneuron within the C-group region.

Finally, the fifth (and lowest dose) injection in this animal (M18-03) consisted of 22 μl of CAV-2-CMV-DsRedII (2.4 × 10^10^ pp) into the left medial rectus muscle (Figure 6A). During surgery, a large bolus was injected into the belly of the muscle, then multiple smaller injections were dispensed as the needle was withdrawn anteriorly. This injection resulted in generally weak labeling in the left oculomotor nucleus (Figures 6B,C). A few labeled cells were also observed just dorsal to the oculomotor nucleus, in the C-group (Figures 6B,C) where MIF motoneurons of the medial rectus reside. There was also labeling within the territory of the SIF medial rectus motoneurons, within the ventral oculomotor nucleus (Figure 6C). Only a few motoneurons were observed, and less dendritic labeling compared to even the lateral rectus injection. Careful morphological inspection of the MIF motoneurons revealed sparse dendritic labeling (Figure 6D).

**FIGURE 7:**
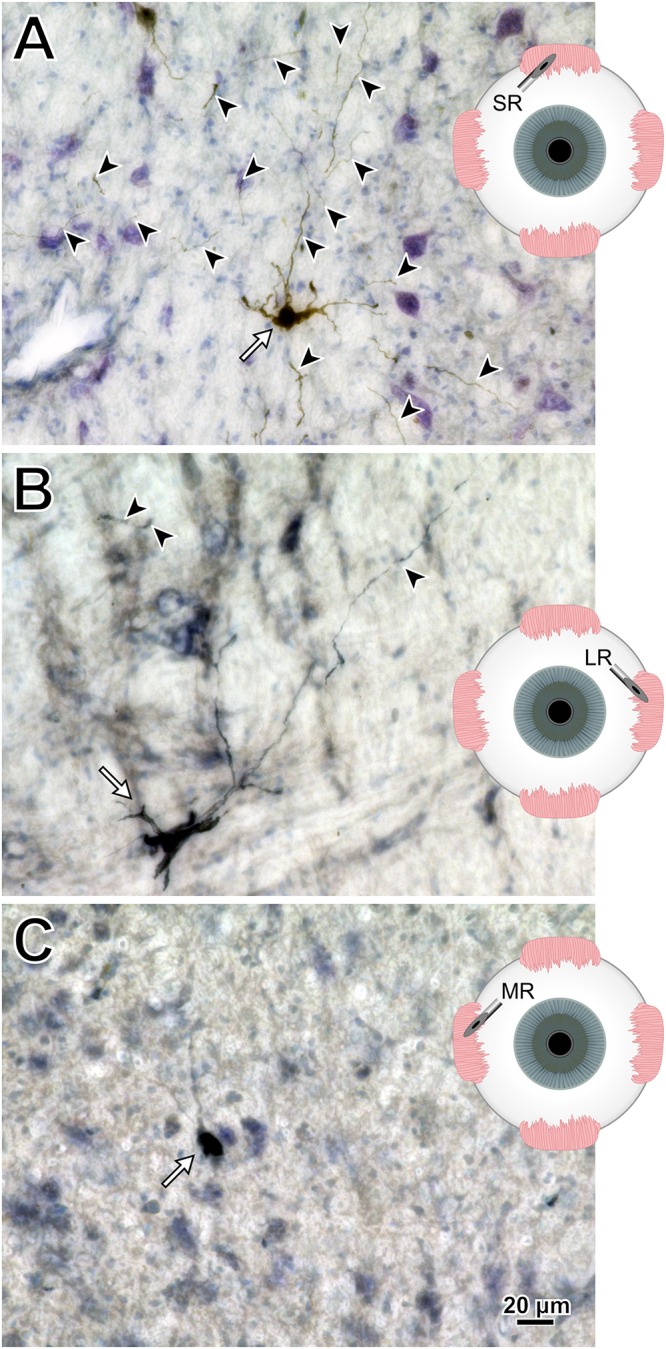
M18-03 photomicrographs of motoneuronal labeling following different CAV-2 dosage injections into the **(A)** superior rectus, **(B)** lateral rectus, and **(C)** medial rectus muscles. Arrows indicate cells, arrowheads indicate dendritic labeling. Photomicrographs **(A–C)** were taken at 40x, A = 25z; B = 1z; C = 2z.

To help compare across injections for this animal (M18-03), photomicrographs in Figure 7 illustrate the quality and intensity of virally mediated labeling of motoneurons innervating the superior rectus muscle (high dose, 7.8 × 10^10^ pp; Figure 7A), lateral rectus muscle (medium dose, 6.2 × 10^10^ pp; Figure 7B), and medial rectus muscle (low dose, 2.4 × 10^10^ pp; Figure 7C). An example superior rectus motoneuron (white arrow; same cell illustrated in Figure 4F) featured numerous short, lightly labeled dendrites (black arrowheads) in the surrounding region (Figure 7A). An example lateral rectus motoneuron (white arrow; same cell illustrated in Figure 5C) had drastically less dendritic labeling in the surround (Figure 7B). Finally, an example medial rectus motoneuron (white arrow; same cell illustrated in Figure 6C) showed no obvious extraneous dendritic labeling (Figure 7C). Across all the CAV-2 injection sites of this case, no gliosis was found histologically in any of the respective nuclei.

Combined, the injections from the third animal (Figures 3–6) revealed an overall diminished degree of labeling, both in the number of cells and dendritic densities, compared to the first animal that only received a single CAV-2 injection (Figures 1, 2). We hypothesized that the differences in motoneuronal labeling were a result of the sum of all CAV-2 injections rather than the titers being injected into individual muscles. To test this, we reduced the number of CAV-2 injections to only two extraocular muscles in a fourth animal (“M19-01”). A general increase in the number of cells and density of dendritic labeling would support the hypothesis. Also, to test whether differences in labeling were dose-related or muscle/nucleus-related, in this fourth animal we injected a higher dose into the medial rectus than into the lateral rectus (opposite to the procedure for M18-03).

**FIGURE 8:**
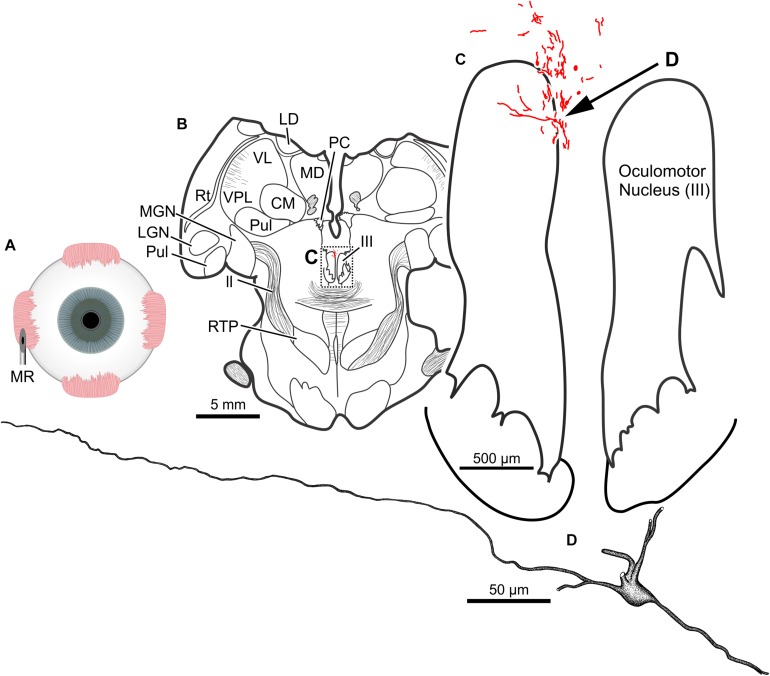
An injection of CAV-2-CMV-DsRedII into the left medial rectus **(A)** resulted in motoneuronal labeling within and dorsomedial to the left oculomotor nucleus. **(B)** Low magnification view of a representative section containing the oculomotor nucleus, box in **(B)** indicates the location illustrated in **(C)**. **(C)** Medium magnification view of the oculomotor nucleus and the distribution of medial rectus motoneuronal DsRedII labeling. Arrow in **(C)** indicates the cell illustrated in **(D)**. **(D)** High magnification reconstruction of a single medial rectus motoneuron demonstrating the extent of motoneuronal labeling provided from an injection of CAV-2.

The results of a 20 μl injection of CAV-2-CMV-DsRedII (1.1 × 10^11^ pp) placed into the left medial rectus muscle of animal M19-01 are shown in Figure 8A. This injection resulted in dense motoneuronal labeling around the left oculomotor nucleus (Figure 8B). A closer inspection of the somatic and dendritic distributions revealed heavy labeling along the dorsomedial edge of the left oculomotor nucleus, the known location of the medial rectus MIF motoneurons (Figure 8C). Most of the dendrites projected ventrally, above the oculomotor nucleus and avoiding the oculomotor nucleus all together. A few dendrites, however, could be observed projecting laterally into the oculomotor nucleus (Figure 8C). In general, labeling was robust enough to visualize somata and primary and secondary dendrites from this injection (Figure 8D).

**FIGURE 9:**
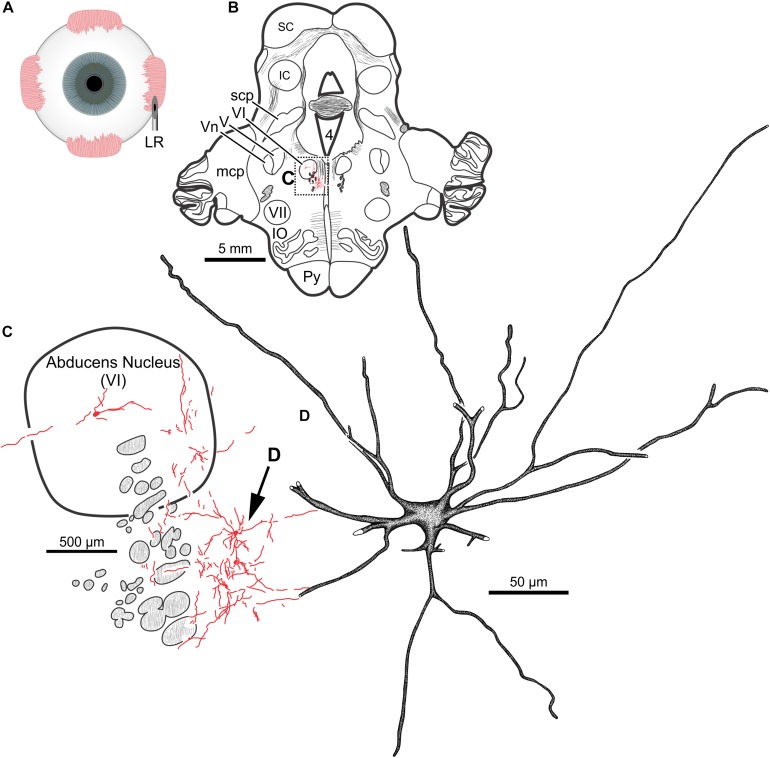
An injection of CAV-2-CMV-DsRedII into the left lateral rectus **(A)** resulted in motoneuronal labeling within the left abducens nucleus. **(B)** Low magnification view of a representative section containing the abducens nucleus, box in **(B)** indicates the location illustrated in **(C)**. **(C)** Medium magnification view of the abducens nucleus and the distribution of labeled neuronal processes. Arrow in **(C)** indicates the cell illustrated in **(D)**. **(D)** High magnification reconstruction of a single lateral rectus motoneuron demonstrating the extent of DsRedII expression within a single motoneuron.

In the same animal (M19-01), 20 μl of CAV-2-CMV-DsRedII (5.6 × 10^10^ pp) was injected into the left lateral rectus (Figure 9A). This injection resulted in motoneuronal labeling within the left abducens nucleus (Figure 9B). Somatic and dendritic labeling was most frequently observed in the periphery of the abducens nucleus (Figure 9C). Close inspection of labeled cells revealed transgene expression throughout the somata and primary, secondary, and tertiary dendrites (Figure 9D).

**FIGURE 10:**
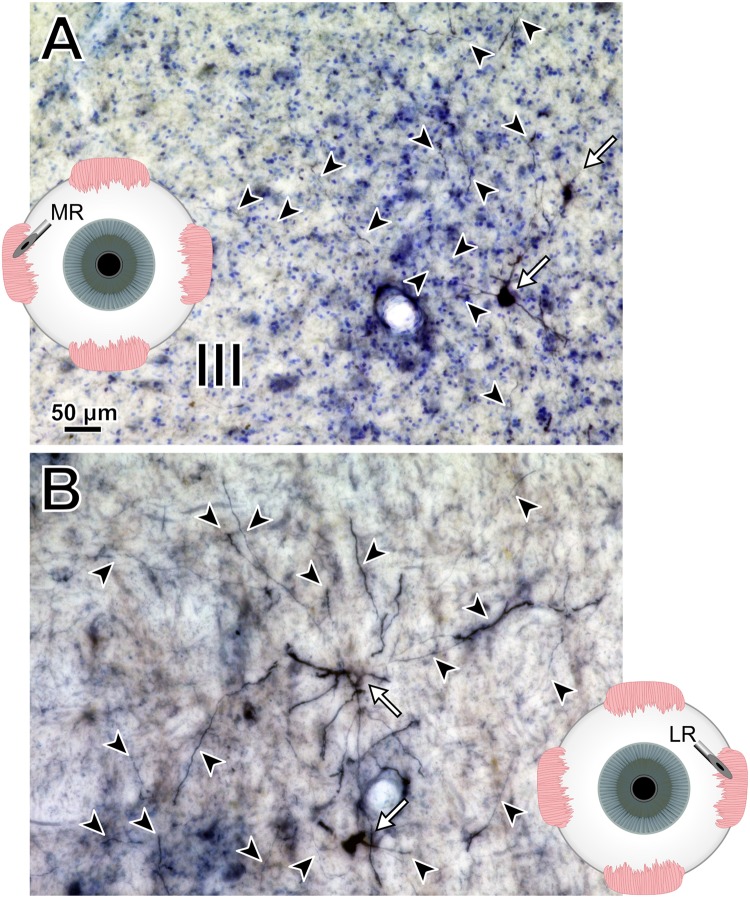
M19-01 photomicrographs of motoneuronal labeling following different CAV-2 dosage injections into the **(A)** medial rectus and **(B)** lateral rectus muscles. Arrows indicate cells, arrowheads indicate dendritic labeling. Photomicrographs **(A,B)** were taken at 20x, A = 1z; B = 25z.

Photomicrographs from the last presented case (M19-01) provide examples of the quality and intensity of CAV-2-mediated expression of DsRedII in medial and lateral rectus motoneurons (Figure 10). Figure 10A is a photomicrograph from the same section as presented in Figure 8, where injections into the medial rectus muscle resulted in well-labeled somata (Figure 10A, arrows) and dense dendritic labeling (Figure 10A, arrowheads) within the C-group around the oculomotor nucleus. Similarly, Figure 10B illustrates the intensity and distribution of labeling within the somata (Figure 10B, white arrows) and dendrites (Figure 10B, arrowheads) of motoneurons in the abducens nucleus. Overall, labeling was denser for the higher titer injection in medial rectus (Figure 10A) compared with the lower titer injection in lateral rectus (Figure 10B). Taken together with the results of animal M18-03, this indicates that labeling was a function of dose rather than the specific muscle/nucleus system.

Though not displayed in the reconstructions, across cases, labeled axons were observed within the fascicules of the oculomotor, trochlear, and facial motor nerves associated with each respective muscle.

## Discussion

The goal of the present work was to evaluate CAV-2’s capacity to transduce exogenous genes into cranial nucleus motoneurons following intramuscular injection in non-human primates. Our histological assessments yielded three main conclusions: (1) The vector worked well to transduce genes into motoneurons of the ocular and facial cranial nuclei (animals M18-01, M18-03, and M19-01); (2) There was an effect of total viral load, with relatively small, single injections yielding the best labeling (M18-01) and larger injections in 2–3 muscles working adequately (M18-03 and M19-01). More extensive injections, however, yielded no transgene expression, presumably due to an immune response triggered by the total viral load (M18-02); (3) With total load constrained to avoid counterproductive immune responses, we found an increasing dose-response relationship over the range of ∼10^10^ to 10^11^ viral particles injected (animals M18-03 and M19-01). We will discuss these results in light of prior results, address the relevance and limitations of the findings, and review important technical considerations when using CAV-2.

### Motoneuronal Labeling Following Intramuscular CAV-2 Injections

The primary finding in this study is that intramuscular injections of CAV-2 reliably transduce cranial nucleus motoneurons in non-human primates. In cranial nuclei that innervate the extraocular musculature, most of the observed labeling was within the territories of MIF motoneurons (located in the periphery of the respective nuclei). We suspect that labeling of the MIF neuromuscular system was favored as a result of our injection approach. The needle was advanced into the muscle at its insertion and driven posteriorly toward the belly. Then, as the needle was withdrawn, small injections of CAV-2 were deposited along its path. Hence, most of the CAV-2 was not in the belly, where SIF neuromuscular junctions reside, but more toward the muscle insertions, where only MIF neuromuscular junctions are found. Correspondingly, we found that transduction was restricted to the relatively small zones in which MIF motoneurons reside, with little if any unintentional encroachment into the larger SIF motoneuron zones. This result provides direct evidence for little, if any, diffusion of the ∼100 nm CAV-2 capsids (Schoehn et al., 2008). If the capsids did not spread longitudinally within the injected muscle toward the belly, it is highly unlikely that they spread to other nearby, intact muscles. In future work, we aim to target both SIF and MIF motoneurons by injecting the central (belly) regions of extraocular muscles to involve the SIF as well as the MIF terminals. We expect that this will result in a drastic increase in the number of motoneurons that are labeled, both by transducing more MIF motoneurons and by adding transduction of SIF motoneurons. This point is substantiated by the medial rectus muscle injection illustrated in Figure 6, in which there was an effort to place more of the CAV-2 injection into the belly of the muscle before releasing virus along the needle track as it was withdrawn; this resulted in labeled motoneurons both within (SIF motoneuron zone) and around (MIF motoneuron zone) the oculomotor nucleus. This is further supported by previous anatomical work in which injections of conventional tracers confined to the insertion of the extraocular muscles resulted in predominantly MIF motoneuronal labeling, while belly injection or full muscle injections resulted in labeling of all motoneuronal populations (Büttner-Ennever et al., 2001; Büttner-Ennever and Horn, 2002). All of this emphasizes the usefulness of the extraocular neuromuscular systems as testbeds for the characterization of viral vectors and the development of viral vector techniques aimed at targeting motoneurons, due to the extraocular muscles’ unique patterns of innervation.

Previous work on transduction of motoneurons following intramuscular injections focused on AAV vectors in spinal systems. Towne et al. (2010) made injections of AAV6-CMV-glb-eGFP into the gastrocnemius muscle of three African green monkeys (*Chlorocebus sabaeus*). Following a 1-month survival period, they found robust fluorescent protein expression in alpha motor neurons within the ventral spinal cord. Using a similar approach, Williams et al. (2018) injected three rhesus macaques with either AAV6-hSyn-ChR2-eYFP or AAV6-hSyn-Chronos-eYFP. Post-injection, optical stimulation of the peripheral nerve produced visually observed fasciculations and electromyographic-detected activity that suggested effective gene expression. Thirteen-weeks post-injection, however, anatomical assessment revealed transgene expression only in the peripheral nerves. A major concern using AAV is that the time course of its expression can be highly variable (Maimon et al., 2017; Williams et al., 2018). More recently, Maimon et al. (2018) found that, in rats, the loss of AAV6-hSyn-ChR2(H124R) mediated protein expression in peripheral nerves was the result of an immunological response against the transgenes. This response resulted in motoneuronal cell death and muscle atrophy. Another downside of AAV6 is that it transduces both the motoneurons and the muscle cells (Towne et al., 2010). This could potentially provide a peripheral catalytic site for developing an immunological response against the transduced exogenous genes. We found strong evidence of CAV-2 transgene expression in motoneurons 2-months post-injection (Table 1), and helper-dependent CAV-2 vectors have been shown to provide constitutive expression over a year post-CAV-2 inoculation (Soudais et al., 2004). In general, CAV-2 shows little immunogenicity in rats, and human sera does not contain neutralizing antibodies (Perreau and Kremer, 2005; Perreau et al., 2007a, b). Further, since the CAV-2 receptor, CAR, is expressed only at the neuromuscular junctions, there is no non-specific uptake of the CAV-2 in the muscle cells; its transduction is restricted to the innervating motoneurons (Soudais et al., 2000, 2001; Salinas et al., 2009). Taken together, a conservative conclusion is that CAV-2 offers a reliable, alternative method to AAV6 for transducing motoneurons following intramuscular injection. With further development of CAV-2 vectors, including helper-dependent approaches, the vector could become a superior tool to AAV6 for basic research studies and gene therapies that target specific motoneuronal populations in non-human primates.

### Dose Responses

A secondary aim of this work was to find an optimal CAV-2 dose that would provide maximal motoneuronal labeling while minimizing cytotoxicity. To this end, there are two dose responses to consider with the current work: the total number of viral particles injected, assessed by comparing across animals, and the viral loads injected into discrete muscles, assessed within animals. First, comparisons across animals suggested that the total number of viral capsids was an important variable (Table 1). The animal receiving the largest vector load, M18-02, received a total of 5.7 × 10^11^ pp but showed no anatomical evidence of transgene expression (Table 2). Animal M18-03 received a total of 4.2 × 10^11^ CAV-2 particles (Figures 3–7), which yielded motoneuronal labeling from all the injection sites, but with relatively weak intensity (Table 2). Animal M18-01 received a single muscle injection with a total of 5.2 × 10^10^ CAV-2 particles (Figure 1), which resulted in robust motoneuronal labeling, but also some gliosis (Table 2). Animal M19-01 received two intramuscular injections for a total of 1.6 × 10^11^ CAV-2 viral particles (Figures 8–10), which yielded moderately strong labeling of both respective motoneuronal populations (Table 2) and no signs of gliosis. In sum, the range from ∼10^10^ to 10^11^ particles yielded the best results. A good example of the total-dose effect is to visually compare the degree of motoneuronal labeling from injections of the orbicularis oculi with relatively higher (Figure 3C) and lower doses (Figure 1C).

With total dose kept in the range ∼10^10^ to 10^11^ pp, allowing for transduction without an apparently counterproductive immune response, we could demonstrate within-animal dose response effects in which the transduction scaled with the dose. The clearest example was for animal M18-03, which received injections of different viral doses into different muscles, with the higher doses yielding better results. Injection of 7.8 × 10^10^ CAV-2 particles into the superior rectus resulted in labeling of the somata, dendrites, and axons of more motoneurons than injection of smaller viral loads into the lateral rectus (6.2 × 10^10^) and medial rectus (2.4 × 10^10^) as summarized in Figures 7A–C, respectively. The difference in labeling seemed related to dose, not muscle or nucleus, because in animal M19-01 we placed the highest dose in the medial rectus (1.1 × 10^11^; Figure 10A) and again it yielded more pervasive labeling than a lower dose, this time placed in lateral rectus (5.6 × 10^10^; Figure 10B). Based upon these results, we suspect that the optimal dose will likely be in the lower range of ∼10^11^ for total intramuscularly injected viral particles; if gliosis results, it should be preventable by dropping the dose to ∼10^10^ while still yielding robust transduction for the muscles tested.

The diminishing returns found for the highest total viral loads are probably related to stronger immune responses mounted in retaliation to the viral insults. While our experiments were not designed to explicitly test immune responses, in at least one case (M18-01; Figures 1, 2), strong transduction co-occurred with clear gliosis as noted above. This could result from an immunological response that was reducing CAV-2 positive neurons, though it is unclear if this was a direct response or a secondary response to potential toxicity resultant from, for example, expression of the fluorophores (Stripecke et al., 1999; Taghizadeh and Sherley, 2008; Ansari et al., 2016). Other vectors have yielded similar results with more direct immunological explanations. For example, previous work with AAV showed that increasing the vector dose improves transduction, but counterintuitively, promotes the activation of T cells (Manno et al., 2006; Mingozzi et al., 2007; Mingozzi and High, 2011, 2013; Nathwani et al., 2011). Overall, the dose effect suggested a complex interplay between the primate immune system and CAV-2. We tested only cranial nucleus motoneuron transduction, and it is unclear whether the optimal dose range suggested by our data generalize to other parts of the central nervous system. For any experiment using CAV-2 *in vivo*, it would seem important to establish dose-response curves to find the optimal dose for safe yet robust transgene expression in the specific, targeted cellular population.

### Technical Considerations

A potential concern with regards to intramuscular injections is the spread of the viral vector from the injected muscle into the surrounding musculature. Based on our observations, this did not appear to be an issue. First, as noted above in §4.1, we found no evidence of spread even longitudinally within the injected muscles. If the capsids do not diffuse a few mm within the penetrated muscle, it is unlikely that they invade neighboring, intact muscles. Any viral spill would need to traverse the orbital space, penetrate the epimysium of another muscle, and then localize to motoneuronal terminals for transduction. Second, our impression was that the immunological response to peripheral viral injections could be stronger in some animals. As would be the case in humans, it is expected to have variable responses across subjects due to the different immunological background of each monkey. Given the volumes injected, the virally mediated labeling described here is far less than is expected from conventional tracer injections of similar volumes (Büttner-Ennever et al., 1981; Porter et al., 1983; Erichsen et al., 2014; Bohlen et al., 2017a). A potential explanation is that the viruses were neutralized soon after being injected into the muscle, and/or that chemical tracers are more readily diffuse throughout the injection site and therefore are capable of being taken up by more neuromuscular junctions. Third, the viral vectors carried different fluorophores that were strategically placed into muscles with neighboring motoneuronal pools. Crosstalk of viruses between the muscles would be noticeable via unexpected labeling in the cranial nuclei. The motoneurons innervating each extraocular muscle reside either in a separate nucleus or, on average, in relatively segregated pools within the same nucleus: lateral rectus motoneurons reside in the abducens nucleus (VI), superior oblique motoneurons reside in the trochlear nucleus (IV), and motoneurons innervating the remaining four extraocular muscles and the levator reside in clustered pools within the oculomotor nucleus (III) with the medial rectus, inferior rectus and inferior oblique motoneurons sitting in pools within the oculomotor nucleus ipsilateral to the muscle they innervate, while the superior rectus motoneurons sit within the contralateral oculomotor nucleus to the orbit the innervate (Evinger, 1988; Büttner-Ennever et al., 2001; Bohlen et al., 2017b, 2019). The instance where this was an issue in the present study was the distinction between CAV-2-hChAT-GFP which was injected into the inferior rectus and CAV-2-CMV-mCitrine which was injected into the superior rectus. The epifluorescence would allow distinction between these two fluorophores, as would labeling of the whole motoneuronal populations. However, the method used for visualization in the present work was immunohistochemical amplification, in which the primary antibody, goat anti-GFP, recognizes both GFP and mCitrine. This left only the anatomical segregation of the two motoneuronal pools. Given that there were GFP positive neurons in the ipsilateral oculomotor nucleus, the known location of the inferior rectus motoneuronal pool, this strongly suggests that CAV-2-hChAT-GFP was able to successfully transduce and express GFP. In the same sections, there was clear motoneuronal labeling both on midline, in the region of the superior rectus MIF motoneurons, as well as labeling in the contralateral oculomotor nucleus (data not shown), a clear sign of superior rectus motoneuronal labeling. Finally, orbicularis oculi motoneurons reside in the dorsomedial division of the facial motor nucleus and masseter motoneurons are found in the trigeminal motor nucleus. Hence, although multiple viruses were injected into multiple muscles of the same orbit, there was no unexpected motoneuronal labeling observed within or across the respective nuclei. These check points provided confidence that a CAV-2 injection into a given muscle produced labeling only in the expected motoneuronal population, and not in others.

Though beyond the current body of work, there is a standing question of labeling with regards to the observed varicosities. It is unclear what these morphological features reflect in terms of the anatomy of labeled motoneurons. Specifically, are the varicosities reflective of bead-on-a-string axons, beaded dendrites, or both? Superficially, the morphological features are akin to the beads-on-a-string frequently observed in tracer-labeled axons, where fine filaments are intersected with boutonal enlargements. These sites of membranous enlargement are presumed to be sites containing vesicles, the molecular machinery necessary for the synaptic vesicular cycle, and anchoring proteins for maintaining pre- and post-synaptic alignment. Further, in the present work, we observed branching patterns that support axonal collateralization (Figure 2B; white filled arrows), where axonal collaterals frequently occur at obtuse or 90° angles and frequently maintain a similar size to the parental stem (Katz, 1985; Peters et al., 1991). In contrast, dendritic branches (Figure 2B; black filled arrow) frequently occur at acute angles and often the branch is smaller than the parental dendrite (Peters et al., 1991). Further, in the best labeled cases, CAV-2 provided fill of axons that could be observed coursing within the fascicles of respective nerves. On the other hand, the presentation of regular ovoid distensions along a dendrite (i.e., beaded dendrites) are assumed to reflect either a problem with the histological preparation or some neuropathology. As it relates to labeling using viruses, Ruigrok et al. (2016) reported gliosis surrounding a subset of rabies positive neurons following injections of rabies into fore- and hindlimb musculature of rats. These neurons appeared to be undergoing degeneration, and accompanying the degenerated neurons were dendrites with a beaded morphological presentation (see Figures 2E1-3 of Ruigrok et al., 2016). Following direct injections of rAAV2-CMV-GFP-TrkB.T1 into the facial motor nucleus, De Wit et al. (2006) also observed some dendritic beading in a subset of the transduced neurons. In the same report, the authors noted that in some cases the dendritic beading occurred throughout all dendrites including primary dendrites coming off the somata (not observed in the current report), while in other examples, beading of distal dendrites but not more proximal dendrites were observed (an occurrence not observed directly in single cells, but which is possible). The Catch-22 is that the cases with the densest labeling/fill were also the cases that, in general, showed the beading morphology and, in a subset of cases, gliosis. We expect that the labeling observed in our sections was likely a mix of axons and beaded dendrites. Fundamentally, more work needs to be done to better understand these morphological features. Future work could use immunofluorescence with antibodies against myelin basic proteins to help determine whether the labeled neuronal structures are axons or dendrites. A more informative option may be to look at the ultrastructure of the labeled neuronal structures. This would not only allow for differentiation of dendritic and axonal structures but also could reveal unique pathological markers.

With regard to the variance in neuronal labeling, two other technical points are notable, as they may be important for optimizing viral vector-mediated transduction and transgene expression in motoneurons: post-injection survival duration and multiplicity of infection (MOI). It is possible that longer survival times could have led to more accumulation of fluorescent proteins that would have become detectable after superseding basal levels of detection. In its simplest form, MOI is a descriptor for the average number of virions infecting each cell (Shabram and Aguilar-Cordova, 2000), in this case, the average number of CAV-2 vectors that successfully transduced motoneurons. A higher MOI should result in overexpression of fluorescent proteins in targeted neurons and with that, an increased probability of immunological detection of the free ends of the CAV-2 genome in the nucleus (Del Rio et al., 2019), likely causing the apparent cytotoxicity. In other words, our results could reflect the low side of what is possible with CAV-2 in cranial motoneurons. The results here provide justification for a broader, systematic parametric study of CAV-2 transduction in macaque cranial nuclei, with the aim of optimizing the ability of CAV-2 vectors to find their receptor and control transgene expression (Perreau and Kremer, 2005; Perreau et al., 2007a, b).

### Challenges and Potential Solutions to the Use of CAV-2 in Macaques

There is potential for CAV-2-mediated gene transfer in non-human primates to be exploited for expression of channelrhodopsin for optogenetics. Although some researchers have observed problems with the expression of channelrhodopsin during CAV-2 vector propagation (personal communication with E. J. Kremer), using neuron-specific promoters may circumvent this (Hirschberg et al., 2017). At present, there is a Cre-expressing CAV-2 that can be combined with a second virus containing a Cre-inducible channelrhodopsin or designer receptor exclusively activated by designer drugs (i.e., DREADDs) that works in primates (Del Rio et al., 2019). However, by requiring a second viral vector, this may decrease the number of neurons that are co-infected with both vectors, resulting in a diminished number of neurons expressing the mature, functional protein. Such an outcome would run counter to one of the main challenges in primate optogenetics: transduction and transgene expression in sufficient numbers of neurons to elucidate full circuits and affect behavior (Galvan et al., 2018). Therefore, the two-vector transactional techniques are less than ideal for primates in which reliable and robust expression is already a challenge.

As noted above in §4.1, one approach that may resolve many of the current issues involving both the immunogenicity and the reliable long-term expression of channelrhodopsin or DREADDs is to develop a helper-dependent (high-capacity, gutted or gutless) CAV-2 vector with the genetic payload of interest. In general, the development of helper-dependent vectors has eliminated many of the immunological issues while also providing constitutive expression over long periods of time in rodents and monkeys (O’Neal et al., 2000; Xiong et al., 2006; Barcia et al., 2007; Butti et al., 2008). More importantly, Soudais et al. (2004) found that a helper-dependent CAV-2 provided transgene expression for over a year without the aid of immunosuppression following intraparenchymal striatal injections in rats. Once a helper-dependent CAV-2 vector has been developed, an important step will be to replicate these findings in primates, requiring that animals survive and are tested at multiple time points for responsiveness to optical or pharmacological stimulation. Here again, the oculomotor system would be a good testbed, as there are established, highly sensitive methods for monitoring eye position in space (Robinson, 1963; Judge et al., 1980). All things considered, CAV-2 is emerging as an important viral vector for primate research, and shows promising clinical potential for its capacity to transduce therapeutic genes to motoneurons following intramuscular injections.

## Data Availability

All datasets generated for this study are included in the manuscript and/or the supplementary files.

## Ethics Statement

The animal study was reviewed and approved by the Duke University IACUC and Institutional Biosafety Committee.

## Author Contributions

MB, HE-N, and MS conceived the work and wrote the manuscript. MB and HE-N performed the surgeries and histology, and analyzed the data.

## Conflict of Interest Statement

The authors declare that the research was conducted in the absence of any commercial or financial relationships that could be construed as a potential conflict of interest.

## Abbreviations

4: fourth ventricle
C: cochlear nucleus
CA: cerebral aqueduct
CAV-2: Canine Adenovirus type-2
Cd: caudate nucleus
CG: central gray/periaqueductal gray
CL: centrolateral thalamic nucleus
CM: centromedian thalamic nucleus
Cn: cochlear nucleus
cp: cerebral peduncle
IC: inferior colliculus III oculomotor nucleus
InC: interstitial nucleus of Cajal
IO: inferior olive
IP: interpeduncular nucleus
IR: inferior rectus
IVn: trochlear nerve
LD: laterodorsal thalamic nucleus
LGN: lateral geniculate nucleus
ll: lateral lemniscus
LR: lateral rectus
M: masseter
mcp: middle cerebellar peduncle
MD: mediodorsal thalamic nucleus
MGN: medial geniculate nucleus
MLF: medial longitudinal fasciculus
MR: medial rectus
ObOc: orbicularis oculi
PAG: periaqueductal gray
PC: posterior commissure
PT: pretectum
Pul: pulvinar
Py: pyramids
RN: red nucleus
Rt: reticular thalamic nucleus
RTP: reticulotegmental pontine nucleus
SC: superior colliculus
scp: superior cerebellar peduncle
SN: substantia nigra
SO: superior oblique
SR: superior rectus
V: trigeminal nucleus
VI: abducens nucleus
VII: facial motor nucleus
VIIn: facial motor nerve
VL: ventrolateral thalamic nucleus
Vn: spinal trigeminal nerve
VPL: ventral posterolateral thalamic nucleus
VPM: ventral posteromedial thalamic nucleus.
